# The Response of Arbuscular Mycorrhizal Fungal Communities to the Soil Environment of Underground Mining Subsidence Area in Northwest China

**DOI:** 10.3390/ijerph17249157

**Published:** 2020-12-08

**Authors:** Hai Huang, Jing Guo, Yuxiu Zhang

**Affiliations:** School of Chemical and Environmental Engineering, China University of Mining & Technology (Beijing), Beijing 100083, China; haihuang@student.cumtb.edu.cn (H.H.); guojing@student.cumtb.edu.cn (J.G.)

**Keywords:** arbuscular mycorrhizal fungi, microbial communities, underground mining, subsidence areas, soil nutrients, enzyme activities

## Abstract

Fully mechanized mining technology applied over a very large working face is typically utilized for coal exploitation in Northwest China and triggered two types of land subsidence above the goaf edge and center. However, the effects of mining subsidence on arbuscular mycorrhizal fungal (AMF) communities are still unknown. Here, we investigated the soil physicochemical properties and the response of AMF communities to the soil environment at the margin and center of the subsidence area of the same working face. Our results showed the soil water content, nutrient content and enzyme activity were significantly decreased with land desertification at the margin of the subsidence area but were less affected at the subsidence center. Utilizing high-throughput sequence analysis, six Glomeromycotan genera were detected. The relative abundance of Glomus and Ambispora at the margin of the subsidence area decreased, while Paraglomus and Diversispora increased. The total OTU richness was significantly correlated with moisture. Redundancy analysis showed the main environmental factors driving the changes in AMF community structure were available nitrogen, available potassium and available phosphorus. All these results indicated land cracks need to be repaired in time at subsidence edge to prevent the decline of soil fertility.

## 1. Introduction

China has the highest coal production worldwide. As coal is excavated, the weight of the overlying strata originally supported by the coal is supported only by the remaining pillars or walls. Subsidence can then occur in the form of ground surface movement or the abrupt creation of a depression in the ground surface, and this leads to geological structure change and even the appearance of cracks [1]. Consequently, land desertification, soil water content and nutrient losses, decreased soil enzyme activities, and soil microbial community changes occur; in particular, the ecological environment in the semiarid desert area of western China has continuously deteriorated as a result of the combination of coal mining and soil erosion. The cracks induced by mining subsidence destroy the soil structure, exacerbate water evapotranspiration, cause severe mechanical damage to plant roots, and cause the ecological environment to become more fragile [2].

Numerous interactions between living organisms and nonliving matter are supported by the soil ecosystem [3]. These interactions are important to ecosystem processes and ecological services. Such interactions include the symbiotic association between most vascular plants and their below ground mycorrhizal symbionts. Arbuscular mycorrhizal fungi (AMF) penetrate the cortical cells of plant roots and maintain symbiotic relationships with most terrestrial plants. These AMF inhabit both plant roots and the surrounding soils and promote the plant’s ability to uptake various mineral nutrients, such as phosphorus and nitrogen. Moreover, the plants involved in AMF symbiosis provide carbon compounds for the fungi while the extracellular hyphae of the AMF provide an expanded root system that assimilates water and nutrients more rapidly from soil areas that are farther away [4]. Because arid and semiarid regions are characterized by low-fertility soils and scarce precipitation that limit plant growth and development, the presence of AMF may be advantageous for mycorrhizal desert plants. Moreover, the previous study by Ng et al. indicated that AM treatments could generally enhance the stele tensile strength, especially the tensile strength of fine stele by AMF symbiosis, enhancing cellulose and hemicellulose biosynthesis [5]. AMF are broadly distributed across different ecosystems, particularly in nutrient-poor ecosystems, such as in coal mining subsidence and arid regions.

However, the vital interactions detailed above are negatively affected by mining and other land use activities [6], which are further reflected in changes in the AMF community structure [7]. Therefore, the differentiation of AMF communities may offer useful insights into the impacts of anthropogenic factors on soil health [8].

Although a number of studies have considered the impacts of coal mining subsidence on bacterial communities [2,9,10], limited research has focused on the impacts of coal subsidence on AMF in semiarid areas. Only Bi et al. have reported on soil AMF in the Shendong Bulianta mining area in Ordos, northern-central China, and their results showed that the AM fungal extraradical hyphal density and phylogenetic index showed a significant decrease in subsidence areas [11]. Surface subsidence is not a homogeneous process. As the mined-out area increases, the range of strata movement increases accordingly. When the area of the goaf expands to within a certain range, movement of the rock layer occurs at the surface, which causes the surface to move and deform. After the mining face stabilizes the surface movement, a sinking basin is formed above the goaf. For horizontal coal seams and rectangular goafs, the final moving basin is elliptical directly above the goaf and symmetrical in relation to the goaf [12]. Moreover, the marginal area has a sloped topography, and surface runoff will carry nutrients and water-soluble ions to the center of the subsidence area [13]. Changes in topography, shifts in moisture and nutrients and differences in plant and microorganism succession will be observed between the center and the margin throughout the subsidence area. Thus, more thorough investigations are needed on the impact of ecological environment degradation in central and marginal subsidence areas.

In this study, the soil properties, enzyme activity and AMF communities at the edge and center of the surface subsidence area in a coal-rich region in the desert of northwestern China were investigated based on the following objectives: (1) to explore the changes in soil quality, enzyme activity and AMF species diversity and differentiation at the margin and center of the subsidence area, (2) to determine the factors that shape AMF assemblages and (3) to obtain insights into the suitability of using AMF community differentiation as an indicator for monitoring ecosystem damage caused by industry and to identify appropriate management practices in the coal mining regions of China.

We tested the hypothesis that the soil in the marginal area of subsidence experiences severe desertification, nutrient losses and decreased enzyme activity and that the AMF assemblages at both the margin and center of the subsidence area differ from those in unmined reference soils. Furthermore, we investigated the influence of environmental factors in shaping soil AMF assemblages in spring and winter which represented the growing period and senescence period of the main plants.

## 2. Materials and Methods

### 2.1. Study Site

**Figure 1:**
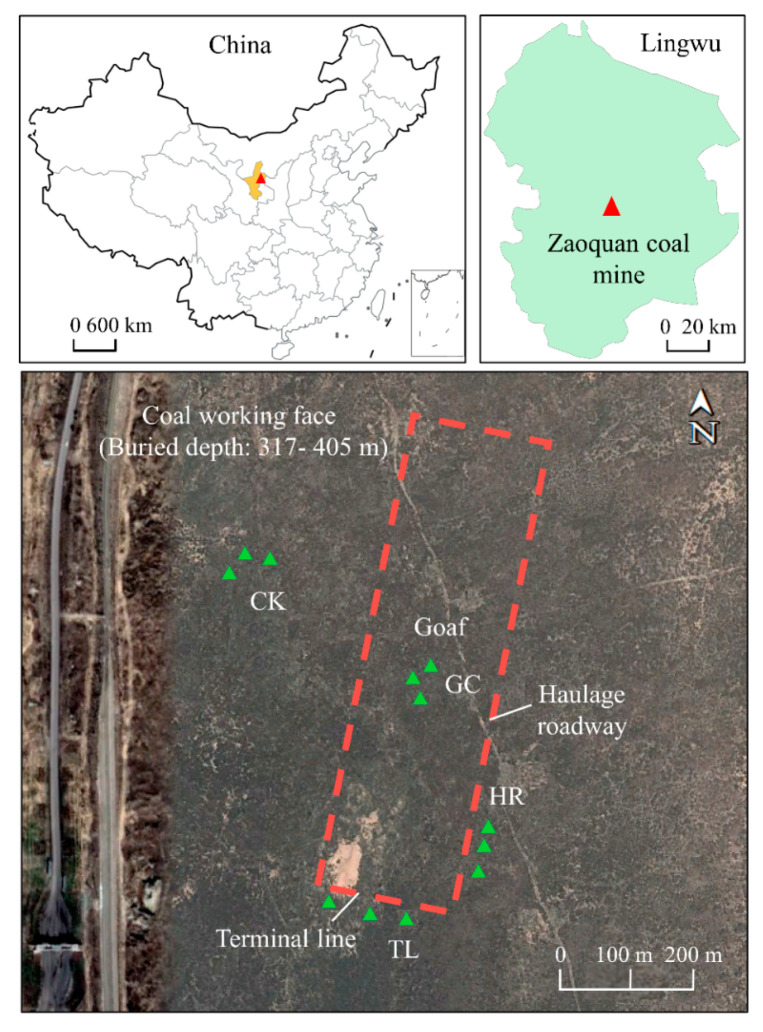
Map of the study site showing the sampling points in the subsidence and unmined areas. The red rectangle represents the approximate position of the working face of the underground coal mine. Each green triangle represents a single quadrat from which samples were collected.

The study site is an underground mining area located in coal-rich Lingwu City, Ningxia, northwestern China (37°52′–38°02′ N, 106°30′–106°35′ E) (Figure 1). This mine field is located on the edge of the Mu Us Desert, 62 km southeast of Lingwu City. The mining area is located in the southwestern corner of the Ordos Plateau, and it is mostly a low-lying landscape with some low mountains. We selected three positions from which to sample soil from the Zaoquan Coal Mine: above the goaf center (GC), representing the even subsidence area; the terminal line area (TL); and the haulage roadway area (HR) at the edge of the coal mining face, representing the uneven subsidence area. Mining in the area ended in 2016, and no reclamation measures were taken after mining (Figure 1). For comparison, an adjacent unmined area was sampled and served as a reference. This adjacent control area (CK) is 200 m away from the mining area and has been rarely subject to human interference. It is worth noting that dense cracks primarily from around the boundary of the mining face instead of at the center, because distortion mostly occurs through horizontal stretching and uneven collapse, which are conducive to the development of cracks (Figure S1). According to local weather station information, the mine field has a semiarid desert continental monsoon climate. The average temperature in summer (June to September) is 22.8 °C, and the average monthly precipitation is 370 mm, while the average temperature in the dry winter months (December–February) is −4 °C, and the average monthly precipitation is 10 mm. The temperature difference between day and night is large. The vegetation cover percent is 10–30%, and the main vegetation type is desert steppe, which is dominated by *Artemisia sphaerocphala.*

### 2.2. Soil Sampling

The growing period of the main vegetation *Artemisia sphaerocphala* is from April to October. Sampling was performed in spring (May) and winter (December) of 2018, which represented the growing period and senescence period of the main plants, respectively. Soil sampling was performed in the four areas (i.e., GC, TL, HR and CK). There was a distance of at least 200 m between the neighboring areas, which had the same soil type and soil texture. In each area, three quadrats (5 m × 5 m per quadrat) were established as triplicate sampling sites (Figure 1). We sampled soils in a vertical direction (0–15 cm and 15–30 cm). In each quadrat, five composite samples were randomly collected at each soil depth. The soil was sieved to remove plant debris, stones, and other non-soil material. Finally, we divided each soil sample into two portions. One portion was air dried and sieved to measure the soil physicochemical properties, and the other portion was kept in a cooler at 4 °C and used to measure the catalase, β-D-glucosidase, urease, and alkaline phosphatase activities and the microbial diversity based on sequencing data.

### 2.3. Soil Physicochemical Analyses and Enzyme Activities

The soil water content (WC) was determined by a gravimetric analysis; briefly, 10.0 g of fresh soil was dried overnight at 105 °C to a constant weight and then the WC was calculated as the difference between the fresh weight and dry weight of the soil [14]. The pH and electrical conductivity (EC) of the air-dried samples were measured using a pH meter and a conductivity meter at 1:2.5 and 1:5 soil/water ratios (*w/v*), respectively [2]. We passed the air-dried soil through a 1 mm sieve, and then the particle fractions were determined using a Mastersizer 2000 instrument (Malvern Instruments, England). Following the taxonomy of the United States Department of Agriculture (USDA), the soil particle size was distributed into silt (2–50 µm), fine sand (50–250 µm), and moderate sand (250–500 µm) fractions. Nitrate (NO_3_^-^-N) was determined following a previous detailed method [15]. Ammonium (NH_4_^+^-N) was analyzed according to the ammonia-selective electrode method [16]. Total nitrogen (TN) was determined following the method of Sparks et al. [17], and soil organic matter (SOM) was determined according to a previously described method [18]. Available phosphorus (AP) and available potassium (AK) were determined using the methods described by Mitchell et al. [19]. The β-D-glucosidase, urease, alkaline phosphatase and catalase activities were determined as previously described [20].

### 2.4. DNA Extraction and Fungal Sequencing

A Fast DNA SPIN Kit for soil was used to extract total genomic DNA from 0.5 g of each soil sample according to the manufacturer’s instructions. An aliquot (25 mL) of extracted DNA from each sample was used as a template for amplification of the hypervariable regions of fungal gene using the nested polymerase chain reaction (PCR) with primers AMV4.5NF 5′-AAGCTCGTAGTTGAATTTCG -3′ and AMDGR 5′-CCCAACTATCCCTATTAATCAT-3′. PCR reactions, containing 25 μL 2x Premix Taq, 1 μL each primer(10 mM) and 3 μL DNA (20 ng/μL) template in a volume of 50 µl, were amplified by thermocycling: 5 min at 94 °C for initialization; 30 cycles of 30 s denaturation at 94 °C, 30 s annealing at 52 °C, and 30 s extension at 72 °C; followed by 10 min final elongation at 72 °C. Three replicates per sample and each PCR product of the same sample were mixed. Length and concentration of the PCR product were detected by 1% agarose gel electrophoresis. PCR products was mixed in equidensity ratios according to the GeneTools Analysis Software (Version 4.03.05.0, SynGene). Then, the mixture of PCR products was purified with EZNAGel Extraction Kit. Sequencing libraries were generated using NEBNext Ultra™ DNA Library Prep Kit (Ipswich, MA, USA) for Illumina. Finally, the library was sequenced on an IlluminaHiseq2500 platform.

### 2.5. Data Analyses

Based on the IlluminaHiSeq2500 sequencing platforms, each sequence was generated from reads of the 250 bp (HiSeq 2500) at the 5′ and 3′ ends using the two-end sequencing paired-end (PE) method. At the same time, sequences were assigned to each sample based on their unique barcode and primer, after which the barcodes and primers were removed and the paired-end clean reads were obtained. The overlapping relationship between PE reads is represented by reads spliced into tags and further filtered to obtain the target tags (clean tags). Quality filtering of the spliced sequences was performed to obtain effective clean tags. Sequences analysis was performed by Usearch software (V8.0.1517). Sequences with ≥97% similarity were assigned to the same OTU (operational taxonomic units), and then OTU species annotation was performed to obtain the community composition information for each sample.

We used unconstrained and constrained criteria to test for relationships among the soil chemical properties, AMF community composition and different soil locations. To analyze the AMF communities between sample groups, the “decostand” function was used to transform relative count data to decimal data in the vegan package (version 4.0) of R software [21]. Comparisons between soil sample groups were based on both the unweighted and weighted Bray-Curtis distance. Furthermore, dissimilarity matrices were generated via principal coordinate analyses (PCoA). Permutational multivariate analysis of variance (PERMANOVA) was used to analyze differences in multivariate space using the vegan package (version 4.0). Post hoc tests of significant PERMANOVA results (*p* < 0.05) were further performed using the “pairwiseAdonis ()” function in the vegan package. The influences of environmental factors, such as soil physicochemical properties and vegetation cover, on the AMF community diversity and structure in both soil and roots were further investigated by Spearman rank correlations and redundancy analyses (RDAs), respectively. The RDA was performed with the log-transformed environmental and AMF community data using an automatic forward and backward stepwise model in CANOCO 5. The significance of the constraining variables was determined based on a permutation test. The effects of site (TL, HR, GC and CK), season (spring and winter) and soil depth (0–15 m and 15–30 cm) and their interaction on soil properties and enzyme activities were tested using general linear models (GLMs). Post-hoc Duncan’s Multiple Range tests were used to compare the means.

## 3. Results

### 3.1. Physicochemical Properties of the Soil

**Figure 2:**
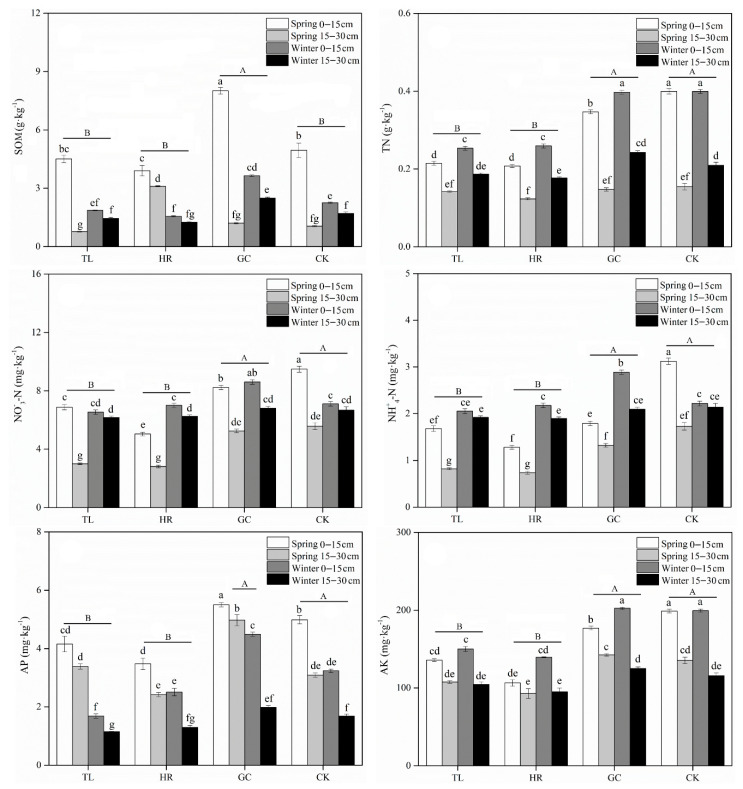
Differences in soil nutrients in the control area and three positions in the subsidence area. Lowercase letters indicate significant differences among samples based on Duncan’s multiple range test (*p* < 0.05) while uppercase letters indicate differences among sites. SOM: soil organic carbon; TN: total nitrogen; TP: total phosphorus; AP: available phosphorus; AK: available potassium.

Soil physicochemical properties were significantly influenced by site, season and soil depth (Table S1). On average, the soil water content (WC) was 0.64–4.27% in spring and 3.18–5.96% in winter. The mean pH of the soil was alkaline and ranged from 7.98 to 8.24 in spring and 7.92 to 8.09 in winter (Table 1). The soil electrical conductivity (EC) in spring (48.92–73.02 μs·cm^−1^) was higher than that in winter (26.15–55.60 μs·cm^−1^). All soil variables, including pH, EC, organic carbon, N forms (NO_3_^-^-N, NH_4_^+^-N and TN), AP and AK, gradually decreased with depth except for the WC, which was highest in the topsoil. The soil moisture and nutrient contents at the margin of the subsidence area (TL and HR) were lower than those in the control area, and the loss of moisture and nutrients in the topsoil was more severe. The soil in the subsidence center (GC) had high WC, EC and nutrients which were close to or even higher than those in the control area (Table 1 and Figure 2). The soil particle size distribution is displayed in Figure S2, which shows that the dominant soil particle type was sand. The silt content was lower at the edge of the subsidence area than in the unmined area, and the sand content was higher. GC was less affected by subsidence and maintained a certain silt content (Figure S2).

### 3.2. Soil Enzyme Activities

**Figure 3:**
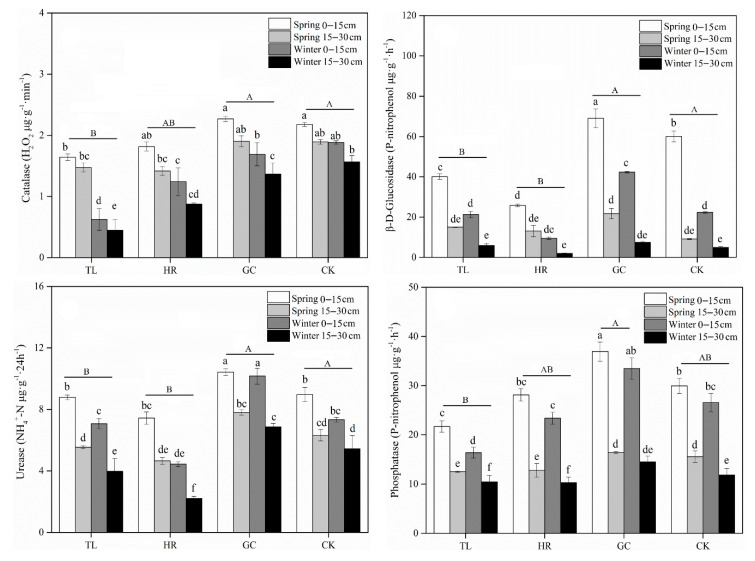
Differences in soil enzyme catalase, β-D-glucosidase, urease, and alkaline phosphatase activity in the control and subsidence areas. Lowercase letters indicate significant differences among samples based on Duncan’s multiple range test (*p* < 0.05) while uppercase letters indicate differences among sites.

Soil enzyme activities were also significantly influenced by site, season and soil depth (Table S2). Soil β-D-glucosidase, urease and alkaline phosphatase activities significantly differed (*p* < 0.05) between the control and subsidence areas (Figure 3). Overall, lower activities of catalase, β-glucosidase, urease and alkaline phosphatase were observed on the subsidence area edge (TL and HR) than in the corresponding reference soils, while the GC soil had high soil enzyme activity values. Furthermore, similar to the distribution of soil nutrients, the enzyme activities in the topsoil (0–15 cm) were higher than those in the deep soil (15–30 cm). In addition, the enzyme activities varied with the season, with generally higher enzyme activity in spring than in winter.

To further explore the relationships between physicochemical properties and enzyme activities, the redundancy analysis (RDA) tri-plot was constructed (Figure S3). The step-wise model for the RDA tri-plot and the first canonical axis (RDA1) of the RDA plot were significant (*p* = 0.04). SOM, AN, AP, and AK were correlated with enzyme activities positively and the sand percentage was the strongest determinant and exhibited a negative relationship with nutrients, and enzyme activities. In addition, for the alkaline soil in the study area, the pH value and enzyme activity were also negatively correlated.

### 3.3. AMF OTU Diversity in Soils

**Figure 4:**
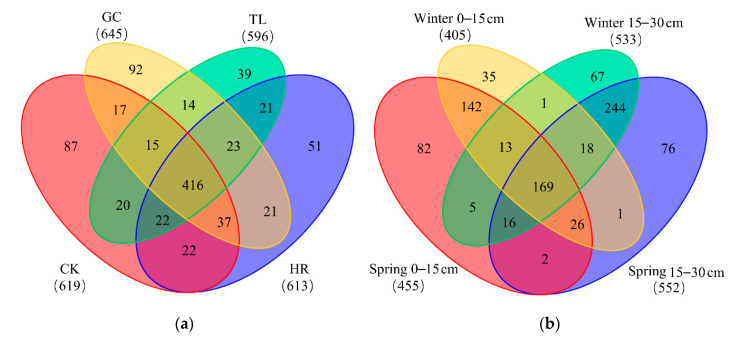
Shared OTUs amongst four sites (**a**) and different seasons and soil depths (**b**). The values in parentheses after each group name indicate the total number of OTUs in the group.

All the rarefaction curves tended to be saturated, indicating that the data volume of sequenced reads was adequate for detecting the majority of sequence types (Figure S4). The number of OTUs in the spring soils was higher than that in the soils collected in winter (Figure 4), and the number of OTUs identified in the deep soil (15–30 cm) was higher than that in the topsoil (0–15 cm). The number of OTUs shared between the spring and winter at the same soil depth represented a high percentage of all OTUs, while the number shared between the different soil depths during the same season was very low. In terms of the unique OTUs at the four sites, GC had the highest number and CK had a higher number than HR and TL (GC > CK > HR > TL). Furthermore, the OTUs shared between HR and TL represented a high proportion relative to their own OTUs and compared to those shared among the two sites and the unmined area (Figure 4).

The observed species number, Chao1 index, and Shannon-Weiner diversity index values for the AMF OTUs in soils at a depth of 0–15 cm were significantly higher than those at a depth of 15–30 cm (Table 2). In addition, these indices of AMF OTUs showed no significant difference between spring and winter and the observed species number and Chao1 index for the soil AMF OTUs differed between the subsidence and control areas. For almost soil samples, the observed species number and Chao1 index values were the lowest in TL and HR, and the observed species number, Chao1 index, and Shannon index of the AMF OTUs were all higher in GC than CK. Except for TL shallow soils, the value of the Shannon-Weiner index in the subsidence areas were higher than that in the control area. Meanwhile, higher dominance tends to appear in deep soil in winter in subsidence areas (Table 2).

### 3.4. Differentiation of AMF Communities in Soils

**Figure 5:**
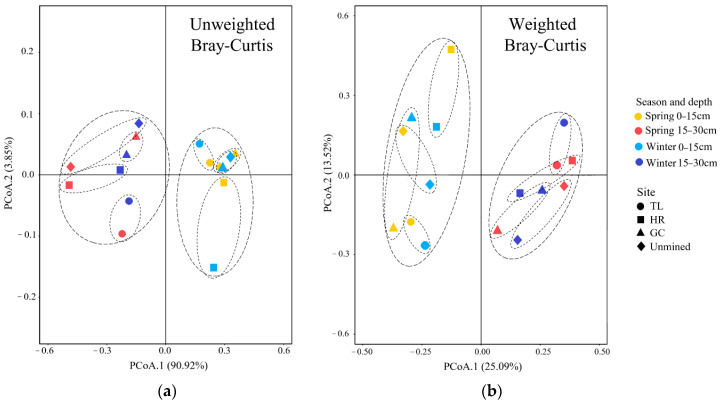
Principal coordinate analysises (PCoA) of the unweighted (**a**) and weighted (**b**) Bray-Curtis dissimilarity among AMF communities. Pair-wise post hoc comparisons for four sites are presented in Table S1.

The Bray-Curtis dissimilarity of the soil AMF community composition (unweighted measure, absence or presence) and structure (weighted measure, relative abundance) (Figure 5) revealed large differences in the soils at depths of 0–15 cm and 15–30 cm (unweighted PERMANOVA R^2^ = 0.634, *p* = 0.001; weighted PERMANOVA R^2^ = 0.352, *p* = 0.002). Close associations were observed in multivariate space between the spring and winter in both the unweighted and weighted PCoA plots of the Bray-Curtis dissimilarities. Pairwise post hoc comparisons of the PERMANOVA results for soils at depths of 0–15 cm and 15–30 cm revealed that the AMF community composition and structure significantly differed (false discovery rate (FDR)-adjusted *p* < 0.05) between the control area and subsidence area (Table S3) as well as between some pairs of subsidence area (Table S2). However, close associations between CK and GC were observed in the top-soils (0–15 cm) with respect to the weighted and unweighted distances (Table S3).

### 3.5. AMF Species Composition and Differentiation

**Figure 6:**
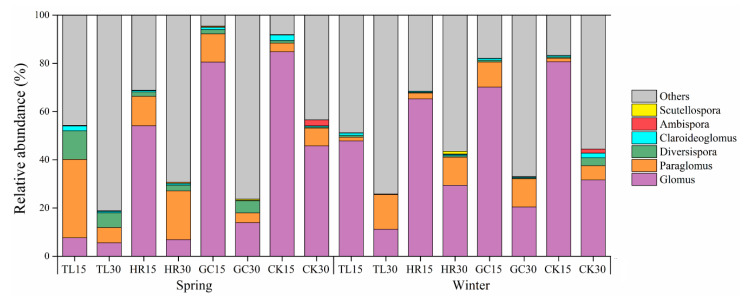
Relative abundances of AMF genera in all samples.

The identified AMF mainly belonged to six families and six genera, namely, *Glomeraceae*: *Glomus*, *Paraglomeraceae*: *Paraglomus*, *Diversisporaceae*: *Diversispora*, *Claroideoglomeraceae*: *Claroideoglomus*, *Ambisporaceae*: *Ambispora*, and *Gigasporaceae*, *Scutellospora*. As shown in Figure 6, the relative abundance of each phylum obviously differed between the topsoil (0–15 cm) and the deep soil (15–30 cm). *Glomus* occupied a dominant position in terms of relative abundance, and decreased in relative abundance at a soil depth of 15–30 cm. The distribution of *Claroideoglomus* in the soil was opposite to that of *Glomus*, with a higher relative abundance in the deep soil. On average, most unclassified sequences at the genus level occurred at a depth of 15–30 cm. In addition, the structure of the soil AMF community differed between the subsidence margin and the control area. The relative abundance of *Glomus* (TL and HR) decreased while that of *Paraglomus* and *Diversispora* increased significantly in the central subsidence area compared to that in the control area. *Ambispora* was associated only with the control area at a soil depth of 15–30 cm, and the relative abundance of *Scutellospora* was the lowest among all genera.

### 3.6. Influence of Environmental Factors on the AMF Community and Species Diversity

Through correlation analysis of AMF community diversity index and soil physical and chemical properties (Table S4), the results showed that OTU richness (Observed species, Chao1) was significantly correlated with WC in spring (Spearman rank correlation, Chao1: r = 0.762, *p* < 0.05) and winter (Observed species: r = 0.738, *p* < 0.05, Chao: r = 0.833, *p* < 0.05). while the value of Shannon-Weiner was negatively correlated with nitrogen forms (TN: r = −0.810, *p* < 0.05, NH_4_^+^-N: r = −0.762, *p* < 0.05 and NO_3_^-^-N: r = −0.810, *p* < 0.05) and pH (r = −0.714, *p* = 0.02) in spring and TN (r = −0.833, *p* = 0.02) and AK (r = −0.881, *p* = 0.02) in winter.

**Figure 7:**
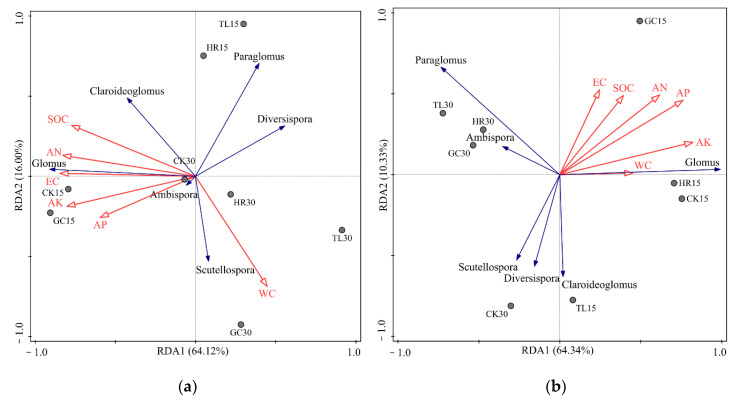
Redundancy analysis (RDA) showing the relationships between soil physicochemical properties and the AMF community in spring (**a**) and winter (**b**). The RDA model for spring and winter was significant (*p* < 0.05). The first axis (RDA1) of the RDA plot was significant in both spring (*p* = 0.032) and winter (*p* = 0.045).

The stepwise model for the RDA tri-plot in Figure 7 was significant (*p* = 0.04) for the AMF community in both spring and winter (*p* = 0.045). In addition, the first canonical axis (RDA1) of the RDA plot for soil was significant for both spring (*p* = 0.036) and winter (*p* = 0.040), and it explained 85.02% and 78.21% of the variation in the AMF community distribution in spring and winter, respectively.

According to the Monte Carlo test (Table S5), the significance of environmental terms fitted into the stepwise RDA model revealed that AN and AK significantly influenced (*p* < 0.05) the AMF community in spring while AN and AP significantly influenced (*p* < 0.05) the community in winter. An analysis of the associations between soil physicochemical properties and the relative percentages (%) of AMF genera in spring (Table S6) revealed significant positive correlations (*p* < 0.05) between *Glomus* and NO_3_^-^-N (r = 0.738, *p* < 0.05), NH_4_^+^-N (r = 0.786, *p* < 0.05), TN (r = 0.810, *p* < 0.05), AK (r = 0.690, *p* < 0.05) and silt (r = 0.857, *p* < 0.01). In contrast, *Paraglomus* was negatively correlated with AP (r = −0.714, *p* < 0.05) and silt (r = −0.833, *p* < 0.05) and *Diversispora* was correlated with NH_4_^+^-N (r = −0.571, *p* < 0.05) and NO_3_^-^-N (r = −0.405, *p* < 0.05). pH was correlated with *Ambispora* (r = −0.738, *p* < 0.05) and *Scutellospora* (r = 0.762, *p* < 0.05). In winter (Table S7), *Glomus* was significantly (*p* < 0.05) positively correlated with NO_3_^-^-N (r = 0.714, *p* < 0.05), AP (r = 0.762, *p* < 0.05) and AK (r = 0.714, *p* < 0.05), *Claroideoglomus* was significantly correlated with pH (r = −0.714, *p* < 0.05) and *Ambispora* was significantly correlated with TN (r = −0.738, *p* < 0.05).

## 4. Discussion

### 4.1. Marginal Mining Subsidence Influenced Soil Physicochemical Properties and Enzyme Activities

Fully mechanized coal mining with a large working face can cause the creation of a sizable goaf as the mining process progresses. Movement of the rock layer occurs at the surface, causing the surface to move and deform, and a sinking basin ultimately forms above the goaf [12]. Dense cracks form mostly around the boundary of the mining face instead of in the center because the surface subsidence around the boundary of the mining face is low while the distortion is mostly horizontal and causes stretching and uneven collapse, which are conducive to the development of cracks [22]. Furthermore, because the topography of the marginal area is sloped, surface runoff will carry nutrients and water-soluble ions to the center of the subsidence area [13]. Therefore, coal subsidence has two different effects at the edge and at the center of the sinking area.

Dense surface cracks may result in the loss of silt and clay, an increase in the percentage of sand, and a tendency for soil to undergo desertification at the subsidence margin. In addition, the cracks destroy the soil structure and increase the total soil porosity, resulting in a decrease in the topsoil water retention capacity. Indeed, the soil WC in the subsidence margin areas was significantly lower than that in the control area. The present study showed that the SOM, TN, AN, and AK contents in the topsoil in the subsidence area significantly decreased, mainly due to the vertical leaching of soil nutrients through the surface fractures. Our results also showed that the vertical distribution of SOM, TN, AP, and AK decreased with depth similar to the results of Yang et al. [23]. An investigation of the distribution characteristics of SOM in subsidence areas associated with coal mining showed that the SOM contents gradually decreased with increasing depth in the soil profile [24]; moreover, all of the selected soil properties decreased with soil depth in the unexploited and sunken areas as the result of the more abundant litter in the top soil and higher permeability, which can promote the growth and metabolism of microorganisms, thereby increasing the nutrient content of the soil during the decomposition process. In addition, the higher EC in the topsoil was attributed to soil water evaporation, which transports salt to the topsoil [2].

Soil enzyme activity is primarily determined by the frequency of substrate–enzyme interactions [25] derived from plant root exudates, soil animals, and especially the soil itself. Soil enzymes are also indicators of soil health and can be used to identify changes in soil biogeochemical cycling potential. Moreover, β-D-glucosidase, alkaline phosphatases, and urease are commonly assayed as indices of C, P, and N cycling, respectively [20], while catalase represents the ability of microorganisms in the soil to perform oxidative decomposition. Enzyme-substrate interactions are controlled by a combination of biological, physical and chemical factors [26]. Because of the more suitable soil temperature and higher metabolism of plants and microorganisms in spring relative to winter, various soil enzyme activities were higher in spring. The chemistry and availability of substrates is also a key factor determining the speed of enzyme activity [27]. The nutrients that had accumulated at the soil surface resulted in the higher quantity and turnover of the enzyme pool caused by the microbial community compared to that in the deeper soil. The average β-D-glucosidase, urease, alkaline phosphatase and catalase activities were lower in the marginal subsidence soils than in the adjacent control soils, which was likely to be due to the considerable loss of nutrient elements in the marginal subsidence area. Importantly, enzyme activities that are sensitive to soil disturbances showed no obvious difference between the central subsidence and unmined areas. The above results indicate that the land collapse weakened the nutrient cycling and oxidation capacity of the soil mainly in the marginal area of the subsidence.

### 4.2. The AMF OTU Richness Decreased at the Margin of the Subsidence Areas

Because of the fragility of desert environments and the frequency of coal mining, AMF have often been proposed as bioenhancers in restoration programs [28]. However, researchers still know little about the genetic and functional diversity of AMF in natural environments. The number of core OTUs shared across all soil samples was larger than that of other groups, indicating similarity in common species at a certain spatial scale. These core OTUs are likely to be influenced by the dominant vegetation species across the coal mining landscape. In addition, the number of OTUs unique to each site was high relative to that of the core OTUs, indicating that subsidence led to a certain degree of species dissimilarity across the landscape. Many studies have shown that coal mining can decrease the abundance of soil microorganisms [2,10,29]. Artificial interference includes agriculture and coal mining, and ecological disturbances have been reported to influence soil AMF richness [30,31]. Similar to these results, AMF richness at the boundary of the subsidence area was lower than that in the unmined area in our study, with a relatively slight trend at the center of the subsidence area. In addition, the higher species dominance in the soil in the unmined area indicates a possible host selection preference for specific species, which could be adapted to the perennials in the surrounding environment. For example, some AMF species have been found to provide plants with more water, phosphorus and nitrogen at the expense of a high carbon drain [32]; therefore, they are less beneficial in the barren and dry soil caused by subsidence.

In terms of large-scale vertical distribution, the number of AMF spores and species decreased with increasing depth [33], which is most likely related to the low organic matter content, availability of oxygen, and the sensitivity of fungi to the low oxygen pressure that occurs in deeper soil layers [13]. For instance, De Araujo Pereira has studied soil AMF in pure and mixed Eucalyptus grandis and Acacia mangium plantations and found that the spore abundance and richness index were significantly decreased in deeper layers (0–20 cm, 20–50 cm and 50–100 cm) [34]. However, another study [35] showed that the richness and diversity of AMF fungi were higher at a soil depth of 15–30 cm than at 0–15 cm, which is similar to the current findings. This can be explained by the fact that the study site has sandy soil and the average thickness of the humus layer is approximately 20–30 cm and shallow soil is loose, porous, and aerated, thus providing enough space for AM fungal sporulation.

### 4.3. Differentiation of Soil AMF Communities in Mining Subsidence Areas

The community composition and structure of AMF assemblages were differentiated in multivariate space, particularly between soils in marginal subsidence areas and unexploited areas. This differentiation suggests the direct impact of soil environment changes on AMF [36], or different colonization needs caused by the adaptability of host plants to soil environment changes. The AMF communities at the center of the subsidence area and the control area soils were similar at a depth of 0–15 cm but different at 15–30 cm, indicating a stronger change in the deep soil environment in terms of craton area. It is worth noting that the soil depth was the most important factor determining the composition and structure of the AMF community in this study, which may have been related to the different physical and chemical properties of the surface and deep soils but also to the morphology of the plant roots. Our results also suggested that the taxonomic composition of the AMF community in soil was fairly stable between spring and winter. Davison et al. suggested that the temporal stability of communities indicates that AMF in soil represent a relatively consistent local species pool from which mycorrhizae form and disband during the growing season [37]. However, plant root samples have demonstrated the seasonality of root colonization by individual members of the AMF community [38,39,40]. In this sense, our results support the idea that natural ecosystems have a fairly consistent soil pool of AMF taxa from which plant–AMF interactions take shape and break apart during the year. Similar to the results of other studies [11,36], the dominant genus in the soil was *Glomus* across the subsidence and unexploited areas. Previous reports have shown that the dominant AMF species will continue to maintain its leading position across a continuous gradient from open sand prairie to closed oak-hickory forest [41].The superiority of *Glomus* in soils may be related to its functional relevance, host-specificity preference, adaptation, and ease of propagation in the soil ecosystem [42,43].

Although the mechanism driving the specific AMF species is unclear, there is evidence demonstrating that plant-specific nutritional requirements are likely to be related to the functional contributions of AMF species in this study [44,45]. However, mining or other man-made disturbances to the land negatively influence the critical interactions between plants and AMF and are further reflected in changes in the AMF community composition [46]. AMF were studied by Melo et al. in multiple soils from pristine natural forests and sites representing a gradient of disturbance, including seminatural and intensively managed pastures in Terceira, Azores, and the results showed that members of *Acaulosporaceae* and *Glomeraceae* were dominant in native forests while members from *Gigasporaceae* and *Claroideoglomeraceae* were most plentiful in seminatural and intensively managed pastures, thus showing family-based ecological preferences [47]. In this study, the relative abundances of *Paraglomus* and *Diversispora* were higher in the subsidence area than the unmined area, demonstrating that these species are more functionally connected with ecological resources and less dependent on the resources provided by plants at specific physiological stages. Almost no *Diversispora* were observed in the soil in spring, which means that some of the AMF species exhibit seasonal differences. By understanding the differences in species relative abundance between collapsed and non-collapsed areas, AMF species that are suitable for use as indicators can be identified for the development of soil ecosystems and can be used as biological fertilizer inoculants in locations affected by different subsidence types for ecological restoration purposes.

### 4.4. Response of the Soil AMF Communities to Changes in Soil Properties

The significant inverse associations between soil AMF species richness and water content is the most critical factor affecting soil microbes in arid environments. Colin focused on elucidating soil responses to seasonal and yearly changes in soil moisture, temperature, and selected soil nutrient and edaphic properties in a sotol grassland in the Chihuahuan Desert and found that changes in soil moisture drive the functioning of soil-microbial dynamics in desert grasslands [48]. Fließbach et al. reported that soils in arid climates typically respond to water pulses with increased soil biological activity, which results in a high respiration rate [49]. The abundance or lack of soil nutrients determines the dependence of plants on AMF, which indirectly affects the AMF community composition in roots and soil. Similar to previous findings [44,50], a negative relationship was observed between the soil nutrients and AMF diversity, which indicates that in the presence of a greater amount of organic matter, AN, and AK, and a relatively high quality of soil, the diversity of AMF species will decrease. Furthermore, environmental factors often affect AMF diversity through the specific regulation of each species.

In the community dimension, the differentiation in soil AMF communities can, remarkably, be explained by the AK, and nitrogen has also been shown to have an effect on AMF communities [36,51]. Total N has been reported to be related to changes in the composition of soil microbial communities [14], and a previous study [51] suggested that N fertilization was the primary factor shaping AMF communities, while another study [52] showed that N addition significantly changed the AMF community structure. A lack of nitrogen can also restrain sporulation, although high N availability can alter nutritional processes in AMF and the abundance of AMF phylotypes. In addition, the N needs of a plant can promote colonization by AMF. The observed influence on fungal community structure indicates that AMF species associated with maples differ in their response to elevated nitrogen. Given that functional diversity exists among AMF species and that N deposition has been demonstrated to result in a decrease in beneficial fungi in some ecosystems, this change in community structure could have implications for the functioning of this type of ecosystem [53]. Moreover, Treseder and Allen observed positive relationships between soil total TN and *Dominikia iranica* (OTU2) and *Rhizoglomus intraradices* (OTU6) [54]. It seems that total N can be a determining factor in improving AMF hyphal length and mycorrhizal biomass as previously reported. In the current study, *Diversispora* was not correlated with AN in winter but was significantly negatively correlated with AN in spring. *Ambispora* also showed a difference in the correlation with total N in different seasons, which is similar to previous findings [55] in which the fungal community response to N deposition was shown possibly to be seasonal. Furthermore, this phenomenon was thought to be attributed to the close connection between plant-specific nutritional requirements and the functional contributions of AMF species [44]. In the growing season, the plant’s demand for external nutrients is often higher than in the non-growing season, leading to a stronger nutrient exchange between plants and fungi, and ultimately resulting in a seasonal response of fungi to changes in the soil environment [56].

Low phosphate (P) availability is a major factor constraining plant growth and metabolism in many soils. P is a vital nutritive element involved in the production of spores by AMF [57]. Significant differences in the community structure of soil AMF were observed between the control and P treatments in the surface soil, and the community shift was mainly attributable to AP, N/P and pH [57]. Few reports have focused on the effect of potassium in the soil on AMF, although a previous study [58] observed that K could limit AMF symbiosis in acidic soil environments with low K [59] because this element is one of the most important inorganic solutes and has a crucial role in many physiological and biochemical plant processes, such as osmoregulation, cell extension, and photosynthesis. It is worth noting that soil pH was not significantly related to AMF community distribution in this study, which is inconsistent with other studies in which pronounced variability in soil pH was shown to have an important effect on AMF community composition [31,60]. This can be explained by the fact that *Glomus* prefers neutral or slightly alkaline soils [61], and this preference might be explained by the generally high soil pH in our study (7.92–8.49), which often leads to AMF communities being dominated by *Glomeraceae* species [7,62], and the lack of a significant correlation between pH and the AMF community.

## 5. Conclusions

At the margin of the coal mining subsidence area, the soil WC, EC, nutrient and enzyme activity at the soil depth of 0–30 cm significantly decreased in association with land desertification, while at the subsidence center, these variables were less affected by subsidence based on a comparison with the unmined area. The AMF assemblages in the unexploited soils were found to vary and differ from those in the soils (0–30 cm) of the subsidence margin and the deep soils (15–30 cm) of the subsidence center. Six *Glomeromycotan* genera were detected in the mine soils, and the relative abundance of the core genus *Glomus* decreased in the marginal subsidence area. Furthermore, the decreasing soil AMF richness was related to the vertical loss of soil moisture and the diversity was influenced by SOM, TN, AN, AP and AK, and AMF structure, specifically driven by AN, AK and AP. This study provides insights into the potentially functionally active species in soil disturbed by mining, which can be adopted for the selective application of AMF species as soil reclamation biofertilizer inoculum.

## Figures and Tables

**Table 1:** Soil physicochemical properties of the samples.

Properties	Season	TL15	TL30	HR15	HR30	GC15	GC30	CK15	CK 30
WC (%)	Spring	0.64 ± 0.03 ^eB^	2.67 ± 0.02 ^dB^	0.66 ± 0.02 ^eB^	2.21 ± 0.03 ^dB^	0.77 ± 0.03 ^eA^	4.27 ± 0.05 ^bA^	0.76 ± 0.02 ^eA^	3.82 ± 0.03 ^cA^
	Winter	3.18 ± 0.02 ^dB^	4.45 ± 0.04 ^bB^	3.78 ± 0.03 ^cB^	4.49 ± 0.03 ^bB^	5.88 ± 0.04 ^aA^	5.96 ± 0.02 ^aA^	5.41 ± 0.05 ^abA^	5.79 ± 0.01 ^aA^
EC (μS/cm)	Spring	60.58 ± 0.35 ^bB^	48.92 ± 0.50 ^cB^	62.28 ± 0.32 ^bB^	52.55 ± 0.56 ^bcB^	73.02 ± 0.23 ^aA^	63.08 ± 0.42 ^bA^	66.05 ± 0.30 ^abB^	57.02 ± 0.38 ^bB^
	Winter	27.35 ± 0.24 ^eB^	26.15 ± 0.34 ^eB^	35.95 ± 0.45 ^dB^	32.10 ± 0.32 ^deB^	55.60 ± 0.42 ^bcA^	54.00 ± 0.44 ^bcA^	39.60 ± 0.34 ^dB^	34.85 ± 0.42 ^dB^
pH	Spring	8.24 ± 0.02 ^a^	8.05 ± 0.03 ^cd^	8.13 ± 0.02 ^b^	7.98 ± 0.01 ^d^	8.07 ± 0.02 ^c^	8.08 ± 0.03 ^bc^	8.17 ± 0.01 ^ab^	8.06 ± 0.01 ^c^
	Winter	8.08 ± 0.03 ^c^	8.09 ± 0.02 ^c^	8.03 ± 0.01 ^cd^	8.04 ± 0.02 ^cd^	7.95 ± 0.01 ^d^	8.13 ± 0.02 ^b^	8.00 ± 0.01 ^cd^	7.92 ± 0.01 ^d^

Values are the means ± SD; Lowercase letters indicate significant differences among samples based on Duncan’s multiple range test (*p* < 0.05) while uppercase letters indicate differences among sites.

**Table 2:** Mean alpha diversity index values for Arbuscular Mycorrhizal AMF communities.

Index	Season	Terminal Line Area (TL)15	TL30	Haulage Roadway Area (HR)15	HR30	Goaf Center (GC)15	GC30	Control Area (CK)15	CK30
Observed species	Spring	334.00	407.00	377.00	403.00	433.00	475.00	394.00	450.00
	Winter	328.00	407.00	343.00	425.00	375.00	458.00	355.00	444.00
Chao1	Spring	404.83	496.17	444.23	470.52	489.71	572.46	451.13	541.96
	Winter	376.30	462.31	408.67	516.56	463.84	541.02	432.04	524.71
Dominance	Spring	0.09	0.03	0.05	0.07	0.10	0.05	0.07	0.10
	Winter	0.14	0.06	0.20	0.05	0.17	0.03	0.10	0.04
Shannon-Weiner	Spring	4.80	5.38	5.31	5.57	5.25	5.48	4.90	5.00
	Winter	3.48	5.10	4.17	5.30	3.99	5.12	3.58	4.82

## Supplementary Materials

The following are available online at https://www.mdpi.com/1660-4601/17/24/9157/s1, Figure S1 Geomorphic pictures of the margin (a) and center (b) of the subsidence area. Huge ground fissures occur at the margin of the subsidence, which results in plant root strain and land desertification. However, the vegetation appears almost unaffected by the collapse of the central area. Figure S2 Soil particle size distribution of samples. Soil particle classifications: silt (5–50 µm); fine sand (50–250 µm); moderate sand (250–500 µm). Figure S3 Redundancy analysis (RDA) showing the relationships between soil physicochemical properties and enzyme activities. RDA model is significant (P < 0.05). The first axis (RDA1) of the RDA plot is significant (P = 0.021) respectively. Figure S4 Rarefaction curve for OTU counts in soil. Table S1 Results of GLMs testing for site (TL, HR, GC, CK), depth (0–15 cm and 15–30 cm) and season (spring and winter) effects on soil properties. Table S2 Results of GLMs testing for site (TL, HR, GC, CK), depth (0–15 cm and 15–30 cm) and season (spring and winter) effects on enzyme activities. Table S3 Pairwise Comparisons of the soil AMF communities. Table S4 Mean alpha diversity index values for AMF communities. Table S5 Significance (Monte Carlo permutation tests) of environmental factors in the reduced RDA model for soil AMF communities in spring and winter. Table S6 Correlation of AMF genera with environmental variables in spring. Table S7 Correlation of AMF genera with environmental variables in winter.
