# Supplementary material for: The Response of Arbuscular Mycorrhizal Fungal Communities to the Soil Environment of Underground Mining Subsidence Area in Northwest China
Source: Int J Environ Res Public Health. 2020 Dec 8;17(24):9157. doi: 10.3390/ijerph17249157 (PMC7763152; doi:10.3390/ijerph17249157)
Supplement: Supplementary file 1 [file ijerph-17-09157-s001.pdf]

Supplementary Materials

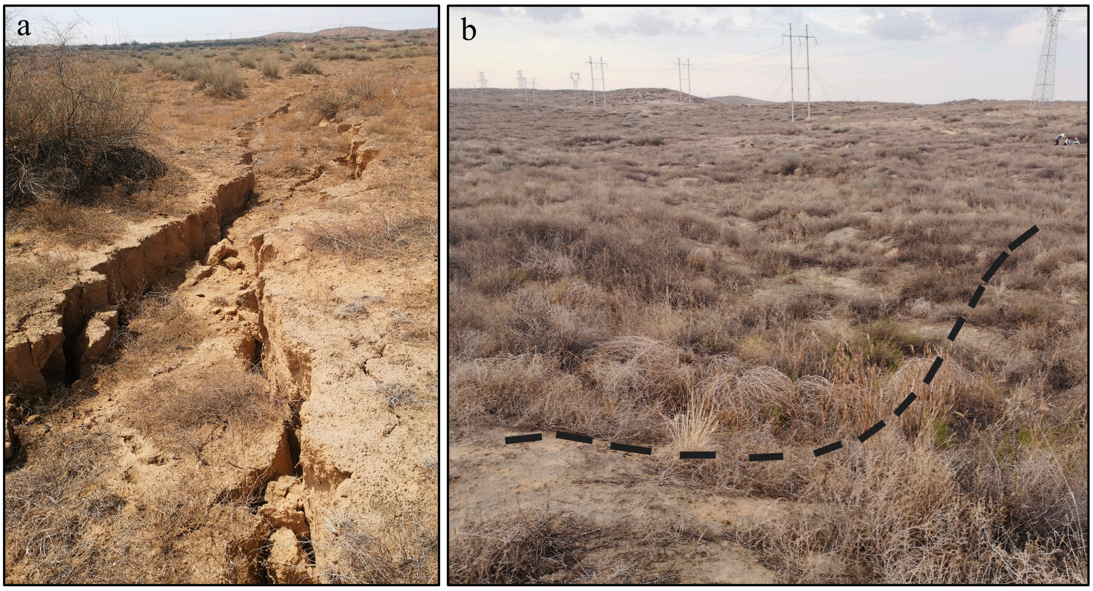

Fig S1 Geomorphic pictures of the margin (a) and center (b) of the subsidence area. Huge ground fissures occur in the margin of the subsidence, which results in plant root strain and land desertification. However, the vegetation appears almost unaffected by the collapse of the central area.

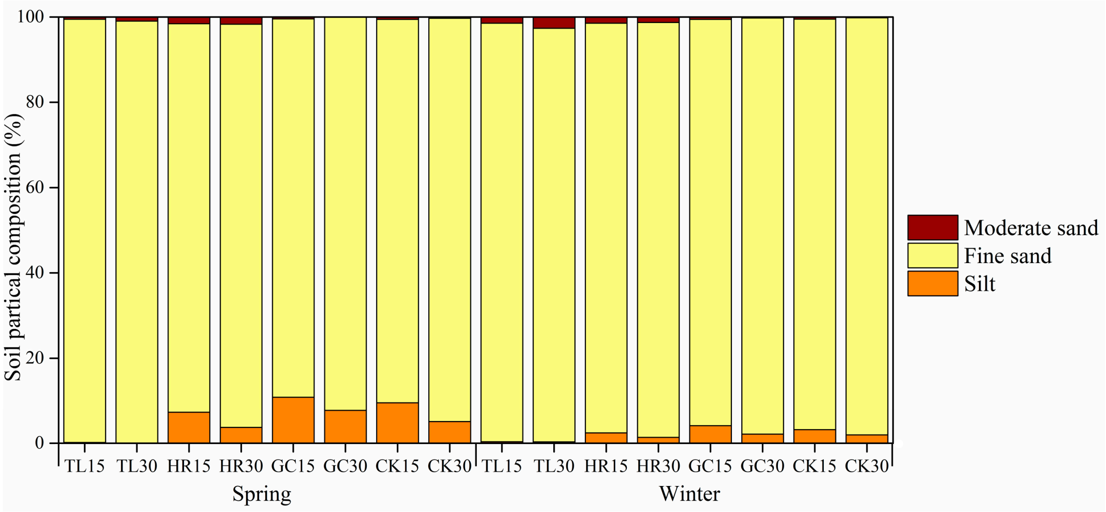

Fig S2 Soil particle size distribution of samples. Soil particle classifications: silt (5-50 μm); fine sand (50-250 μm); moderate sand (250-500 μm).

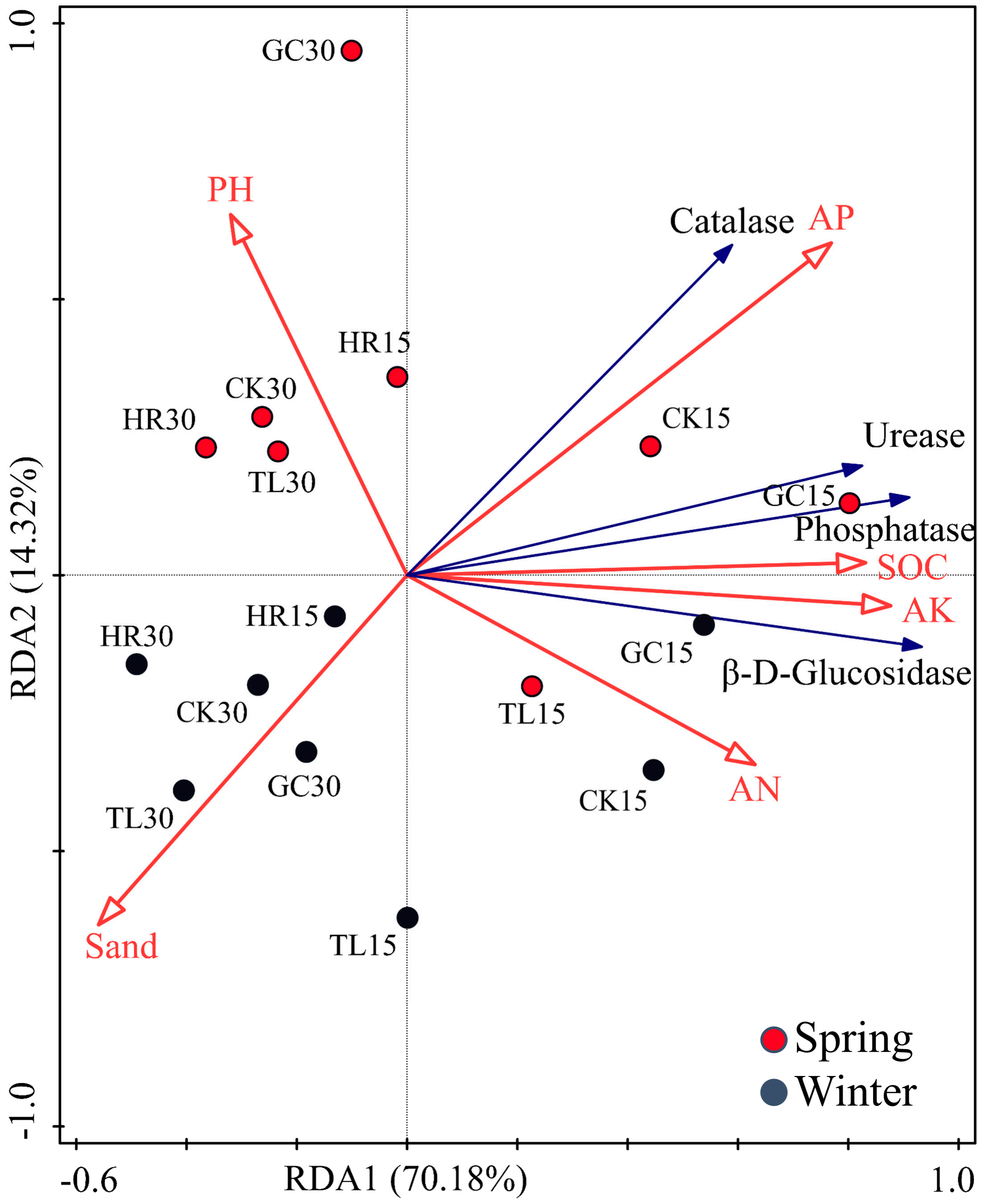

Fig S3 Redundancy analysis (RDA) showing the relationships between soil physicochemical properties and enzyme activities. RDA model is significant ($P < 0.05$). The first axis (RDA1) of the RDA plot is significant ($P = 0.021$) respectively.

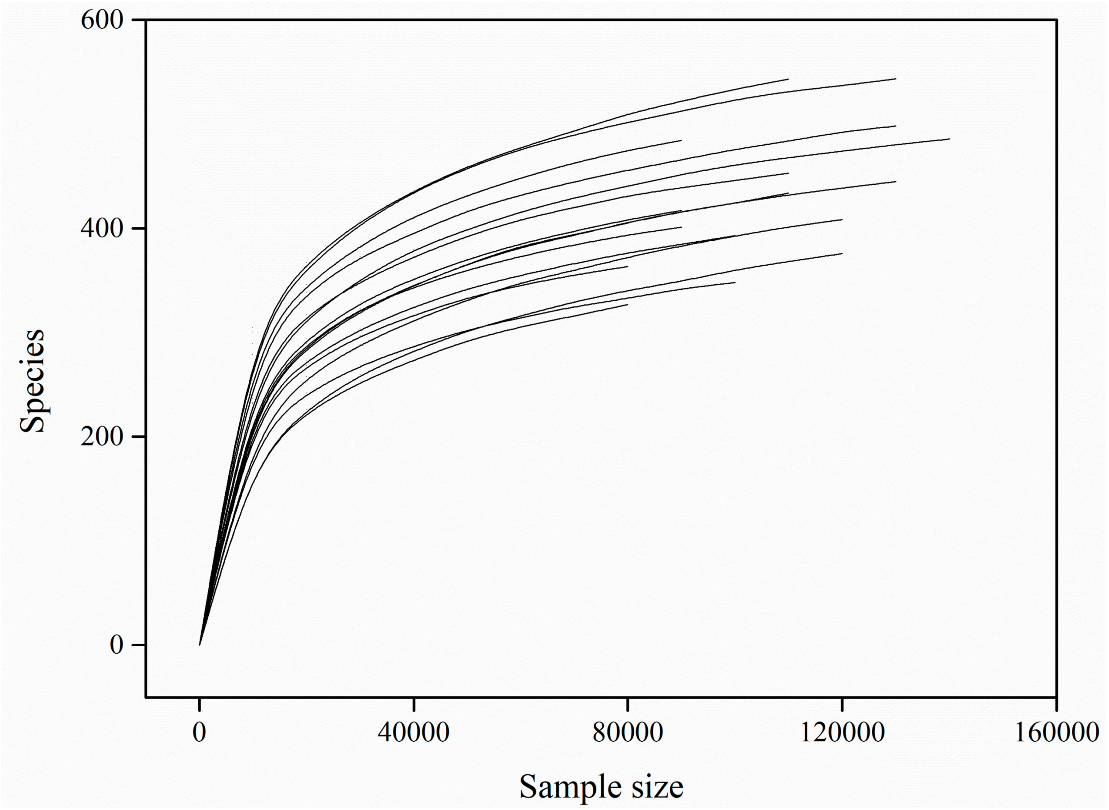

Fig S4 Rarefaction curve for OTU counts in soil.

Table S1 Results of GLMs testing for site (TL, HR, GC, CK), depth (0-15 cm and 15-30 cm) and season (spring and winter) effects on soil properties.

Source	WC（%）	EC（μS/cm）	pH	SOC	TN	$N0_3^-$-N（mg/kg）	NH_4^+-N（mg/kg）	AP（mg/kg）	AK（mg/kg）
	P	P	P	P	P	P	P	P	P
Site	0.035*	0.023*	0.176	0.046*	0.032*	0.029*	0.024*	0.012*	0.032*
Depth	＜0.001*	＜0.001*	0.058	＜0.001*	0.002*	＜0.001*	＜0.001*	＜0.001*	＜0.001*
Season	＜0.001*	＜0.001*	0.067	0.281	0.007*	0.087	0.037*	＜0.001*	0.129
Site × Depth	0.033*	0.187	0.287	0.274	0.067	0.012*	0.064*	0.032*	0.067
Site × Season	0.052	0.267	0.522	0.117	0.121	0.047*	0.079*	0.056	0.198
Depth × Season	0.079	0.127	0.238	0.016*	0.023*	0.043*	0.017*	0.025*	0.012*
Site × Depth × Season	0.045*	0.154	0.342	0.098	0.087	0.032*	0.231	0.034*	0.089

*Significant at $P < 0.05$.

Table S2 Results of GLMs testing for site (TL, HR, GC, CK), depth (0-15 cm and 15-30 cm) and season (spring and winter) effects on enzyme activities.

Source	catalase （H_2O_2 μg·g^{-1}·min^{-1}）	β-D-glucosidase（P-nitrophenol μg·g^{-1}·h^{-1}）	urease（NH_4^+-N μg·g^{-1}·24h^{-1}）	alkaline phosphatase（P-nitrophenol μg·g^{-1}·h^{-1}）
	P	P	P	P
Site	0.012*	0.034*	0.042*	0.023*
Depth	＜0.001*	＜0.001*	＜0.001*	＜0.001*
Season	0.002*	0.002*	0.001*	＜0.001*

Site × Depth	0.017*	0.087	0.023*	0.012*
Site × Season	0.079	0.042*	0.062	0.007*
Depth × Season	0.026*	0.167	0.008*	0.002*
Site × Depth × Season	0.056	0.087	0.043*	0.006*

*Significant at P < 0.05.

Table S3 Pairwise comparisons of the soil AMF communities.

Depth	0-15 cm				15-30 cm			
Bray-Curtis dissimilarity	Unweighted		Weighted		Unweighted		Weighted	
Site Pairs	P-value	P (FDR-adjusted)	P-value	P (FDR-adjusted)	P-value	P (FDR-adjusted)	P-value	P (FDR-adjusted)
Unmined vs. TL	0.026*	0.038*	0.017*	0.035*	0.012*	0.038*	0.018*	0.036*
Unmined vs. HR	0.014*	0.026*	0.014*	0.026*	0.021*	0.043*	0.021*	0.034*
Unmined vs. GC	0.122	0.143	0.068	0.842	0.031*	0.042*	0.024*	0.039*
TL vs. HR	0.012*	0.032*	0.005*	0.022*	0.013*	0.037*	0.027*	0.034*
TL vs. GC	0.028*	0.045*	0.021*	0.045*	0.008*	0.038*	0.017*	0.039*
HR vs. GC	0.012*	0.036*	0.016*	0.034*	0.021*	0.042*	0.026*	0.032*

*Significant at P < 0.05. FDR-adjusted, false discovery rate-adjusted. Pairwise comparisons were performed using the "PairwiseAdonis" function in the vegan package of R software.

Table S4 Mean alpha diversity index values for AMF communities.

Season	Spring	Winter

	Observed species	Chao	Dominance	Shannon-Weiner	Observed species	Chao	Dominance	Shannon-Weiner
WC	0.690	0.762*	0.060	0.357	0.738*	0.833*	-0.524	0.214
EC	-0.143	-0.286	-0.048	-0.024	-0.119	0.000	0.357	-0.119
PH	-0.524	-0.571	0.084	-0.714*	0.119	0.000	-0.286	0.333
TN	-0.333	-0.452	0.422	-0.810*	-0.571	-0.452	0.619	-0.833*
NH_4^+-N	0.071	-0.048	0.602	-0.762*	-0.262	-0.119	0.476	-0.619
NO_3^--H	-0.071	-0.190	0.554	-0.810*	-0.214	-0.048	0.429	-0.571
AP	0.190	0.095	0.301	-0.548	-0.357	-0.19	0.524	-0.667
AK	0.024	-0.095	0.096	-0.405	-0.571	-0.429	0.619	-0.881**
SOM	-0.333	-0.500	0.349	-0.500	-0.024	0.119	0.095	-0.595
Silt	0.381	0.238	0.157	0.000	-0.095	0.071	0.357	-0.405

*Significant at $P < 0.05$; **Significant at $P < 0.01$

Table S5 Significance (Monte Carlo permutation tests) of environmental factors in the reduced RDA model for soil AMF communities in spring and winter.

	Spring				Winter		
	Explanatory %	pseudo-F	P		Explanatory %	pseudo-F	P
AN	33.2	8.8	0.016	AK	27.6	5.8	0.04
AK	22.2	6.1	0.04	AP	24.9	4.6	0.047
SOM	10.1	1.9	0.218	SOC	7.7	2.7	0.166
EC	9.2	1.5	0.256	AN	7.7	2.4	0.192
AP	3.2	0.5	0.62	EC	3.2	1.5	0.33
WC	1.1	<0.1	0.894	WC	3.6	5.5	0.24
pH	1.1	<0.1	1	pH	0	<0.1	1

Table S6 Correlation of AMF genera with environmental variables in spring.

	Glomus	Paraglomus	Diversispora	Claroideoglomus	Ambispora	Scutellospora
WC	-0.548	0.167	0.143	-0.714	0.643	-0.405
EC	0.571	-0.286	0.095	0.119	-0.452	0.571
pH	0.429	0.024	0.095	0.381	-0.738*	0.762*
TN	0.810*	-0.405	-0.381	0.595	-0.476	0.452
NH^{+}_{4}-N	0.786*	-0.571	-0.571*	0.405	-0.214	0.310
NO^{-}_{3}-N	0.738*	-0.500	-0.405*	0.524	-0.310	0.429
AP	0.619	-0.714*	-0.262	0.381	-0.381	0.595
AK	0.690*	-0.690	-0.095	0.381	-0.548	0.714*
SOM	0.762	-0.357	-0.190	0.619	-0.452	0.548
Silt	0.857**	-0.833*	-0.643	0.024	-0.286	0.333

*Significant at P < 0.05; **Significant at P < 0.01. Spearman correlation in spring was performed on the log-transformed species relative count and environmental data.

Table S7 Correlation of AMF genera with environmental variables in winter.

	Glomus	Paraglomus	Diversispora	Claroideoglomus	Ambispora	Scutellospora
WC	-0.048	0.000	0.190	0.262	0.381	-0.048
EC	0.119	0.214	-0.190	-0.238	-0.357	0.333
pH	-0.524	0.500	-0.667	-0.714*	-0.167	0.333
TN	0.810*	-0.69	0.095	0.262	-0.738*	-0.048
NH_4^+-N	0.667	-0.500	0.190	0.333	-0.429	-0.262
NO_3^--N	0.714*	-0.476	0.143	0.262	-0.500	0.024
AP	0.762*	-0.524	0.119	0.238	-0.619	0.119
AK	0.714*	-0.619	0.238	0.381	-0.619	0.000

SOM	0.286	-0.238	0.167	0.286	-0.310	0.143
Silt	0.690	-0.381	0.095	0.19	-0.405	0.071

*Significant at P < 0.05; **Significant at P < 0.01. Spearman correlation in winter was performed on the log-transformed species relative count and environmental data.
